# Multi-Omics Characterization of a *KIF1C* Structural Variant in a Patient with a Complex Movement Disorder Partially Responsive to Deep Brain Stimulation

**DOI:** 10.1007/s12311-026-01963-x

**Published:** 2026-03-24

**Authors:** Mirja Thomsen, Max Borsche, Vicente A. Yépez, Dirk Rasche, Kristian K. Ullrich, Vera Tadic, Saad M. Abdelwakeel, Hauke Busch, Sören Franzenburg, Joanne Trinh, Christine Klein, Katja Lohmann, Norbert Brüggemann

**Affiliations:** 1 Institute of Neurogenetics, University of Lübeck, Lübeck, Germany; 2 Department of Neurology, University of Lübeck, Lübeck, Germany; 3 School of Computation, Information and Technology, Technical University of Munich, Munich, Germany; 4 OmicsDiscoveries GmbH, Planegg, Germany; 5 Department of Neurosurgery, University of Lübeck, Lübeck, Germany; 6 Division Scientific IT Group, Max Planck Institute for Evolutionary Biology, Plön, Germany; 7 Emergency Department, University Hospital of Schleswig-Holstein, Campus Lübeck, Lübeck, Germany; 8 Medical Systems Biology Group, University of Lübeck, Lübeck, Germany; 9 School of Biotechnology, Nile University, Giza, Egypt; 10 Institute of Clinical Molecular Biology, Kiel University and University Hospital Schleswig-Holstein, Campus Kiel, Kiel, Germany

**Keywords:** Ataxia, KIF1C, Optical genome mapping, Quadruple DBS, Gene dosage change, Spastic ataxia type 2 (OMIM: 611302), Dystonia, Myoclonus, Head tremor, Spasticity, Leukoencephalopathy, Hard-to-detect variant, Transcriptome, Rare neurological disease

## Abstract

Biallelic *KIF1C* variants are associated with a spectrum of neurological phenotypes, including spasticity, cerebellar ataxia, chorea, and dystonia. Most reported patients involve single-nucleotide variants, whereas copy number variants (CNVs) have been described only once in the PubMed-listed literature. The therapeutic potential of deep-brain stimulation (DBS) in *KIF1C*-related disease has not yet been evaluated. We report a patient presenting with spasticity, ataxia, dystonia, and head tremor, in whom prior exome sequencing was inconclusive. A multi-omics approach was applied, including optical genome mapping (OGM), genome sequencing, and transcriptome sequencing. The clinical effect of DBS was assessed. OGM revealed a homozygous *KIF1C* Exon 17–18 deletion, retrospectively detectable in exome and genome data but initially missed by sequence analysis. Transcriptome sequencing demonstrated significantly reduced *KIF1C* expression and absence of normally spliced transcripts around those exons, consistent with a loss-of-function mechanism. DBS led to partial clinical improvement, with notable reduction of dystonia and head tremor. Structural variants may be an underrecognized cause of complex movement disorders involving spasticity, ataxia, and dystonia. Diagnostic methods specifically targeting structural variation, such as OGM or CNV analysis of short-read data, can be essential for diagnosis. Transcriptome data add valuable functional insight. This report also suggests that DBS may offer symptomatic benefit in *KIF1C*-associated disease, particularly for dystonic signs.

**Supplementary Information:**

The online version contains supplementary material available at 10.1007/s12311-026-01963-x.

## Introduction

The recent development of molecular genetic methods has opened a new era of diagnosing rare neurogenetic diseases, enabling the identification of causes in previously molecularly undiagnosed patients [1]. Among these methods, short-read exome and genome sequencing are particularly effective in detecting single-nucleotide variants (SNVs) and small insertions or deletions (indels), while advanced bioinformatic tools have improved the identification of other variant types, such as copy number variants (CNVs), albeit with limitations [1–3]. In contrast, Optical Genome Mapping (OGM), a technique that utilizes ultra-long, fluorescently labeled DNA molecules, is specifically suitable to detect large structural variants (SVs) without sequencing [4]. Furthermore, the integration of other omics technologies, such as transcriptome analysis, may guide variant interpretation [1]. 

These developments may also impact studies on hereditary ataxias and hereditary spastic paraplegias (HSP) [5] that are characterized by a plethora of genetic alterations and complex phenotypes with varying clinical and genetic overlap [6]. For example, biallelic variants in the *KIF1C* (*Kinesin Family Member 1 C*) gene cause an autosomal recessively inherited complex movement disorder, referred to as spastic ataxia type 2 (SPAX2) or HSP/ATX-KIF1C[7–9]. *KIF1C* encodes a microtubule-dependent motor protein essential for intracellular transport [10–12]. Only about 25 patients with biallelic *KIF1C* variants have been reported thus far in the PubMed-listed literature, including truncating[8, 9, 13], splice site[8, 14, 15],, and missense variants[9, 16], as well as one four-exon deletion [8]. 

Here we characterize a biallelic 2-exon deletion in a patient with an infantile-onset, complex movement disorder partially responding to deep-brain stimulation (DBS), identified through OGM and further analyzed using exome, genome, and transcriptome data.

## Methods

After consenting to the research project, the patient underwent video-taped neurological examinations and biomaterial collection. The Ethics committee of the University of Lübeck approved the study, according to the Declaration of Helsinki.

### Optical Genome Mapping (OGM)

Ultra-high-molecular-weight (UHMW) genomic DNA (gDNA) was extracted from fibroblasts using the Bionano Prep SP-G2 and labeled using the DLS-G2 Protocol. The labeled DNA was loaded onto a Bionano Saphyr Chip, linearized in nanochannels, and imaged with the Saphyr instrument. The run yielded 606.75Gbp of fragments > 150kbp with 169× effective coverage, 90.4% map rate, N50 of 347.29kbp, and label density of 14.47/100kbp. De novo assembly was performed with Bionano Solve (v3.8.1), and SVs were analyzed in Bionano Access (v1.8) using the T2T-CHM13 reference genome.

### Exome and Genome Sequencing

Genomic DNA was extracted from peripheral blood using the QIAamp DNA Mini Kit (Qiagen). Exome and genome sequencing were performed at the Competence Centre for Genomic Analysis (CCGA), Kiel, Germany. For exome sequencing, the Illumina DNA Prep with Enrichment kit and IDT xGen Exome v2 baits were used. Whole-genome sequencing libraries were prepared using the Illumina DNA Prep Kit. Sequencing was performed on an Illumina NovaSeq 6000 with 150 bp paired-end reads, achieving 99.8× coverage for the exome and 52.7× coverage for the genome of the index patient. Reads were aligned to the hg38 reference genome using BWA-mem2.

### RNA Sequencing

Total RNA was extracted from peripheral blood using the PaxGene Blood RNA kit (Qiagen). Library preparation was done with the Illumina Stranded Total RNA Kit, and sequencing was performed on an Illumina NovaSeq 6000 at CCGA, Kiel, Germany. RNA-seq data were aligned using the nf-core rnaseq pipeline v3.15.1 with the STAR aligner (v2.7.10a) and Trim Galore (v0.6.7) for read trimming. Quality control was performed using Qualimap (v2.3), RseQC (v5.0.2), SAMtools (v1.2), and MultiQC (v1.24.1). Aberrant expression and splicing were detected using DROP’s modules, based on OUTRIDER and FRASER 2.0 methods [17]. To increase statistical power, 347 blood samples from Solve-RD were added to the cohort. 

## Results

**Fig. 1:**
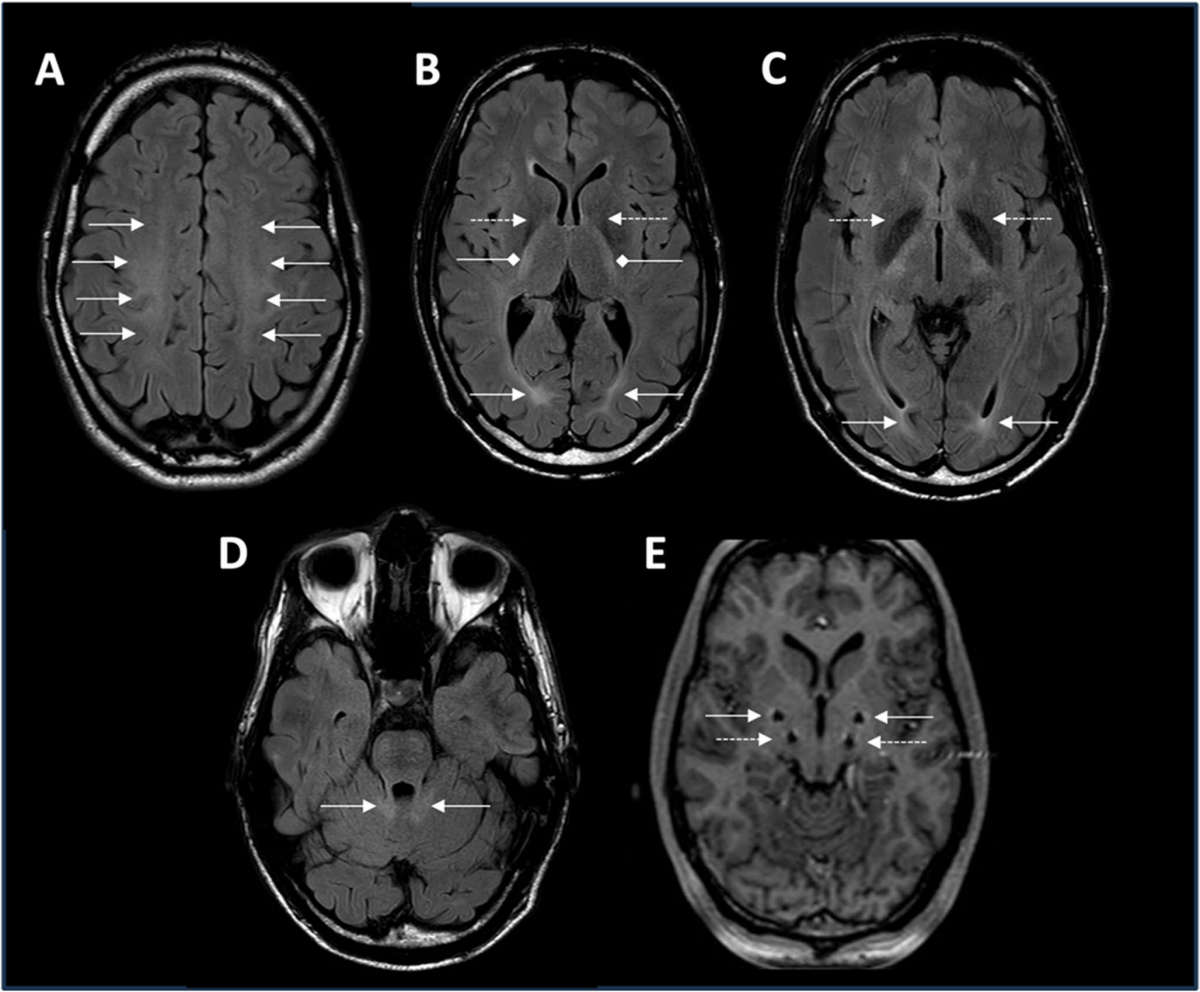
MR imaging. **A-D**: MRI from the reported patient at the age of 27 years. Axial T2-weighted (fluid-attenuated inversion recovery) images are shown. Imaging demonstrated symmetrical T2-hyperintense cerebral demyelination involving central (white arrows in **A**) and occipital (white arrows **B** and **C**) white matter, the internal capsule (white square-ended arrows in **B**), and the cerebellar peduncles (white arrows in D). Moreover, symmetric T2 hypointensities in the globus pallidus were observed (white dotted-line arrows in **B** and **C**). **E.** Post-surgery MR imaging of the four deep brain stimulation electrodes bilaterally in the globus pallidus internus (GPi, white arrows) and nucleus ventralis intermedius (ViM, white dotted-line arrows)

We report a White male patient with a predominantly hyperkinetic and progressive movement disorder manifesting immediately after birth with flaccid muscular tone. At the age of 2 years, abnormal head posturing and tremor were noticed and worsened over the following 6 years, particularly when writing. Afterwards, the symptoms were more slowly progressive. Upon neurological examination in his early twenties, he exhibited head tremor, dystonia predominantly of the neck and trunk, cerebellar ataxia with severe intention tremor and dysarthria, spastic paraparesis, and myoclonic jerks (Video 1). Clinical features also include mild horizontal saccadic pursuit, intermittent nystagmus, as well as proximal weakness of both legs (grade 3 on the Medical Research Council [MRC] scale for muscle strength). Intake of alcohol resulted in an improvement, especially of myoclonic jerks. The patient had no cognitive impairment and successfully graduated from law school. The family history was negative, but his parents were consanguineous, being second-degree cousins. MRI showed symmetric white matter T2-hyperintensities and bilateral hypointensities of the globus pallidus (Fig. 1A-D).

Propranolol (40 mg per day at the age of 20 years) had a transient effect on tremor and myoclonus, whereas baclofen (dosage unknown), L-Dopa (300 mg), and carbamazepine (300 mg) did not lead to relevant clinical improvement. Trihexyphenidyl in a daily dosage of 15 mg had a moderate effect on the tremor and cervical dystonia. Although the patient suffered from a multisystemic neurological syndrome, a treatment attempt with DBS was initiated at the age of 25 years after thorough consultation targeting dystonia, tremor, and myoclonus through stimulation of the globus pallidus internus (GPi) and nucleus ventralis intermedius (ViM) bilaterally, before the genetic cause was determined. DBS improved cervical and trunk dystonia and myoclonic jerks subjectively by 80% ten months post-surgery, with improved head control and purposeful grasping, relevantly ameliorating the patient’s quality of life. Objective measurements of dystonia severity or quality of life using rating scales and questionnaires, respectively, were not performed prior to DBS. Dysarthria deteriorated slightly at 19-month follow-up and improved again at 31-month follow-up, while the other symptoms remained stable over this period. The optimal clinical effect was achieved with GPi stimulation, allowing ViM stimulation to remain discontinued at the last follow-up. The stimulation parameters were as follows: left GPi: 8–, 6.5 mA, 60 µs, 180 Hz, right GPi: 4–, 6.5 mA, 60 µs, 180 Hz. The patient was last evaluated at the age of 34 years, nine years after DBS surgery, demonstrating a persistent, marked improvement in cervical dystonia and myoclonus, while the tremor of the upper extremities showed only a moderate response (Video 2). A slight worsening of the gait disturbance was observed. The patient lives independently and is employed in a regular administrative position. Trihexyphenidyl had an additional symptomatic effect, after a dosage increase of 6–10 mg/day at 19-month follow-up and 15 mg/day at the latest follow-up. Table 1 provides an overview of symptoms at onset and the chronological evolution of symptoms before and after DBS surgery over the disease course. Table 2 presents the DBS parameters accompanied by the respective co-medication at follow-up visits.

**Table 1:** Symptoms at onset and chronological evolution of symptoms before and after DBS surgery

Age at onset	• 2 years • mild motor developmental delay noted previously, with milestones achieved within the normal age range
Symptom evolution before DBS	• 2 years: Abnormal head posturing and tremor • 3 years: Single febrile seizure • 8 years: Tremor of upper extremities, particularly during writing • School age: Use of a typing device; attendance at a school for children with physical disabilities despite normal intellectual development • Puberty: Marked clinical deterioration with onset of spasticity and foot drop • Thereafter: Very slow progression • 23 years (first presentation): Able to walk short distances; otherwise wheelchair-dependent, symptoms worsen with caffeine intake and show marked improvement with alcohol
Symptom evolution after DBS	• 25 years: DBS of the ventral intermediate nucleus (VIM) and globus pallidus internus (GPi), activation of GPi DBS • 10 months follow-up: subjective improvement of cervical/truncal dystonia and myoclonic jerks by ~ 80% • 19-month follow-up: deterioration of dysarthria • 28-month follow-up: additional programming of ViM DBS without benefit (discontinued after weeks) • 31-month follow-up: improvement of dysarthria, with stable control of other symptoms • 34 years (last clinical follow-up): persistent marked improvement in cervical dystonia and myoclonus; moderate response of upper extremity tremor, progression of lower-limb weakness and spasticity with worsening of gait

**Table 2:** Evolution of DBS parameters and accompanying co-medication at follow-up examinations

Time point	Left GPi	Right GPi	Left ViM	Right ViM	Co-medication
3-month FU	10-, monopolar, 125 Hz, 3.0 V (= 2.8 mA), 90 us	5-, monopolar, 125 Hz, 3.0 V (= 3.3 mA), 90 us	Discontinued	Discontinued	-
10-month FU	10-, monopolar, 125 Hz, 4.0 V (= 3.6 mA), 90 us	5-, monopolar, 125 Hz, 4.0 V (= 3.9 mA), 90 us	Discontinued	Discontinued	Trihexyphenidyl 6 mg/die
19-month FU	10-, monopolar, 125 Hz, 4.1 V (= 3.7 mA), 90 us	5-, monopolar, 125 Hz, 4.1 V (= 3.9 mA), 90 us	Discontinued	Discontinued	Trihexyphenidyl 10 mg/die
28-month FU	10-, monopolar, 125 Hz, 4.1 mA, 90 us	5-, monopolar, 125 Hz, 4.1 mA, 90 us	2 + 3-, bipolar, 125 Hz, 3.7 mA, 60 us	14 + 15-, bipolar, 125 Hz, 3 mA, 60 us	Trihexyphenidyl 15 mg/die
31-month FU	10-, monopolar, 125 Hz, 4.1 V (= 3.6 mA), 90 us	5-, monopolar, 125 Hz, 4.1 V (= 4.1 mA), 90 us	Discontinued*	Discontinued*	Trihexyphenidyl 15 mg/die
9-year FU	8-, monopolar, 180 Hz, 6.5 mA, 60 us	4-, monopolar, 180 Hz, 6.5 mA, 60 us	Discontinued	Discontinued	Trihexyphenidyl 15 mg/die

DBS settings (Medtronic Activa RC) are displayed as DBS contact, polarity, frequency, amplitude, pulse width, GPi – Globus pallidus internus; ViM – Nucleus ventralis intermedius; FU – follow-up; * Deactivated by the patient between 28- and 31-month FU, no additional benefit

### Identification of a Homozygous *KIF1C* Deletion

**Fig. 2:**
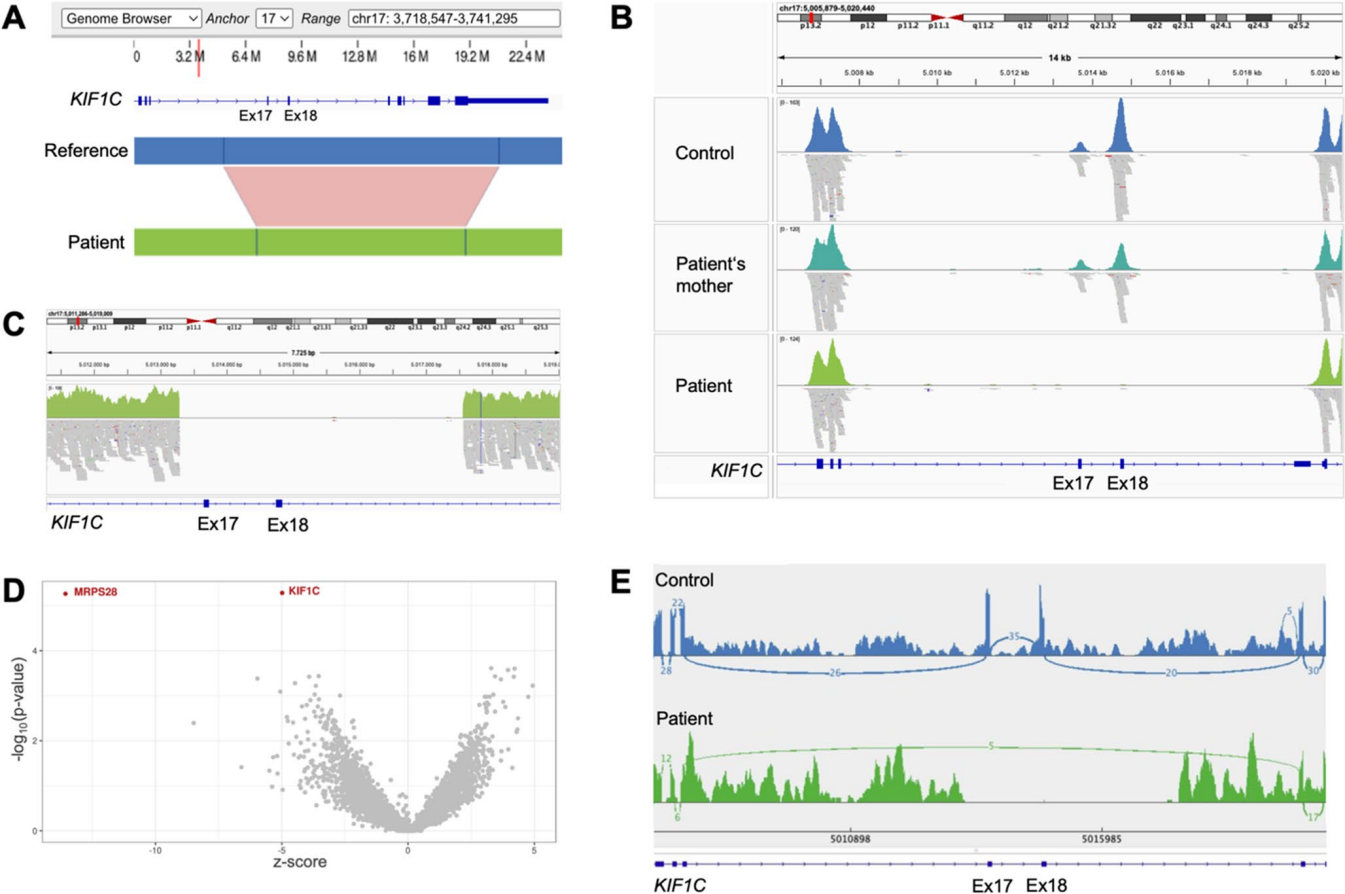
Genomic and transcriptomic analyses. **(A)** Optical Genome Mapping detected a homozygous deletion at chr17:4,901,429–4,915,093 (T2T reference genome, corresponding to chr17:5,011,105–5,024,768 in the hg38 reference) in the patient, which is within the *KIF1C* gene. **(B)** Integrative Genomics Viewer (IGV) snapshot of exome sequencing coverage for *KIF1C*. The patient shows no reads covering Exons 17–18, consistent with a homozygous deletion. The mother shows reduced coverage (~ 50%) of these two exons compared to a control sample (upper panel), indicative of a heterozygous deletion. **(C)** IGV snapshot of genome sequencing reads revealing a homozygous 4,275 bp deletion with precise breakpoints (hg38; chr17:5,013,289–5,017,563). **(D)** Volcano plot from OUTRIDER-based expression outlier analysis, revealing *KIF1C* as a significantly underexpressed gene in the patient. The x-axis shows the z-score of gene expression, and the y-axis indicates the –log₁₀(p-value). **(E)** Sashimi plot of the *KIF1C* transcript from patient RNA sequencing data shows absent expression of Exons 17–18, with evidence of exon skipping (Exon 16 directly spliced to Exon 19)

Using OGM, a homozygous deletion of an estimated 4,245 bp within a 13,665 bp region on chromosome 17, encompassing Exons 17–23 of the *KIF1C* gene (NM_006612.6), was identified in the patient (Fig. 2A). Prior clinical trio-exome sequencing, in 2016, which had focused on SNVs and small indels, had been negative. Next, to determine the exact exonic region affected by the deletion, exome sequencing reads from a new sequencing run in 2021 were inspected using the Integrative Genomics Viewer (IGV). This was needed since OGM only estimates an interval and the size of a structural variant based on label distance and cannot determine the exact breakpoints. This analysis revealed absence of sequencing coverage for Exons 17–18 in the patient, while the surrounding exons were adequately covered (Fig. 2B). Exome data from the unaffected mother showed reduced coverage of these exons (~ 50%) compared to a control sample, consistent with a heterozygous deletion. The father is presumed to be heterozygous for the deletion as well, given the consanguinity and homozygosity in the patient, but his carrier status could not be assessed. Further examination of the patient´s genome sequencing data generated in parallel with OGM confirmed the precise breakpoints at NC_000017.11:5013289_5017563del (hg38), corresponding to NM_006612.6:c.1492_1666del, representing a 4,275 bp deletion (Fig. 2C). This deletion is absent in the gnomAD CNVs 4.1.0 database.

### Reduced *KIF1C* Expression and Aberrant Splicing

RNA sequencing confirmed *KIF1C* disruption, identifying it as an expression outlier in the patient (p-value = 5.19 × 10^− 6^, z-score = −4.99, fold change = 0.73) (Fig. 2D), indicating significantly reduced mRNA expression by 27% compared to the mean of other analyzed transcriptomes. IGV inspection of RNA sequencing data revealed no expression of Exons 17–18 (Fig. 2E). Additionally, *KIF1C* was identified as a splicing outlier (p-value = 2.50 × 10^− 8^), with a complete absence of normal splicing of those two exons. Further, IGV showed several split reads in which Exon 16 was directly spliced to Exon 19 (Fig. 2E).

On the protein level (NP_006603.2), skipping of Exons 17–18 is predicted to result in a frameshift starting at amino acid residue 498, creating a premature stop codon at position 512 (p.Thr498Trpfs*15). Additionally, the *MRPS28* gene, encoding Mitochondrial Ribosomal Protein S28, was identified as an expression outlier in the patient (p-value = 5.43 × 10^− 6^, z-score = −13.56, fold change = 0.07) (Fig. 2D). However, no SNVs, indels, or structural variants based on genome sequencing and OGM data were found that could account for the reduced expression. Moreover, the signal was based on an unusual read distribution lacking clear splice junctions and including reads across intronic regions, suggesting a technical artifact (e.g., misalignment or RNA degradation).

## Discussion

Here, we describe a biallelic 2-exon deletion in *KIF1C* in a patient with an infantile-onset, complex movement disorder, partially responsive to DBS. The structural variant was first identified through OGM, with complementary tools required to determine the precise location. The genetic diagnosis initially escaped detection through exome sequencing, as the analysis focused on SNVs and small indels. Although CNV calling was not initially performed on exome data, advanced CNV-calling tools, such as ExomeDepth, later also revealed the homozygous deletion in the patient, but not the heterozygous one in the mother, likely because it only comprised 2 exons. These findings underscore the need for broader application of comprehensive CNV detection tools in patients with complex syndromes, including ataxia and spasticity.

Genetic analyses suggest that the deletion of Exons 17–18 results in partial nonsense-mediated mRNA decay (NMD), as evidenced by lower *KIF1C* expression levels in the patient, based on transcriptome analysis. This only modest reduction (–27%), despite biallelic loss-of-function, likely reflects both the low baseline expression in blood and incomplete degradation of the mutant transcript by NMD. Residual mRNA levels in peripheral blood may underestimate the full extent of the transcriptional impact in neural tissues, where *KIF1C* is strongly expressed, and the effect is expected to be more pronounced.

Further, the deletion is expected to introduce a frameshift and a premature stop codon, likely leading to a complete absence of functional KIF1C protein. If any protein were produced, it would be missing more than half of its sequence (amino acids 498-1,103), encompassing crucial domains involved in autoinhibition and interaction with binding partners [18]. Taken together, we consider this biallelic multi-exon deletion to be disease-causing, as it results in a clear loss-of-function of KIF1C, consistent with the established biallelic loss-of-function mechanism underlying *KIF1C*-related disease.

Previously reported disease-causing variants in *KIF1C* include missense variants in the kinesin motor domain, splice site, and truncating variants, as well as a deletion of Exons 14–18 in a Moroccan family [8] – a deletion overlapping with the one found in our patient. Identifying another SV in *KIF1C* highlights the need to consider this variant type in genetic diagnostics, particularly when only a heterozygous pathogenic SNV is found, as it may be combined with a heterozygous CNV.

While ataxia and spasticity are the core symptoms in *KIF1C*-associated disease, our patient’s additional clinical features, i.e., dystonia, head titubation/tremor, and characteristic distribution of leukoencephalopathy, conform to the few *KIF1C*-linked disease patients previously published [9, 16]. In detail, dystonia was also reported in 5 of 20 (25%) patients with homozygous *KIF1C* variants (Supplementary Table 3). Of note, in patients without large exon deletions, a later age at onset [9, 16] and less pronounced MRI alterations [9] have been reported, compared to our patient with an exonic deletion, suggesting a potential genotype-phenotype correlation. However, such interpretations are limited by the very small number of patients with *KIF1C*-associated disease and by the lack of detailed published phenotypic data. Importantly, there is no report on DBS in *KIF1C*-related disease yet, probably because DBS cannot be expected to have an ameliorating effect on cerebellar ataxia or spasticity. Nevertheless, our patient underwent DBS before the genetic diagnosis was made to improve the patient’s prominent dystonic and myoclonic symptoms (by bilateral electrodes in the GPi) and tremor (by bilateral electrodes in the ViM). He benefited from DBS, although the therapeutic response was difficult to anticipate given the complex phenotype and neuroimaging abnormalities. GPi DBS was effective in improving dystonia and myoclonic jerks, whereas ViM DBS did not provide additional benefit for tremor control and was therefore not pursued. The at least partial response of the patient’s dystonic symptoms is in line with the well-established beneficial effect of GPi-DBS in many genetically caused generalized dystonias caused by pathogenic variants in e.g., *TOR1A*,* SGCE*, and *VPS16* [19, 20]. 

In conclusion, the clinical benefit observed after DBS highlights a potential symptomatic treatment option in *KIF1C*-linked disease, particularly in patients presenting with dystonia, myoclonus, and head tremor. At the same time, this report underscores the importance of including CNV analysis in the genetic workup of complex movement disorders, as well as the value of a multi-omics approach – in this case, additional transcriptome analysis that provided insights into the deletion’s effects on mRNA expression and splicing.

## Supplementary Information

Below is the link to the electronic supplementary material.

Supplementary Material 1 The video shows the patient nine years after DBS surgery, demonstrating an almost normal head position, reduced myoclonus, and improved intention and action tremor. The stimulation parameters at that time were as follows: left GPi: 8–, 6.5 mA, 60 μs, 180 Hz, right GPi: 4–, 6.5 mA, 60 μs, 180 Hz (MP4 198 MB)

Supplementary Material 2 The video features the patient at age 25 years prior to DBS implantation. Head and neck dystonia, head tremor, dysarthria, postural, action, and intention tremor, and intermittent myoclonic jerks, mostly at the upper half of the body, are shown. At the end of the video, the patient shows severe spastic gait disturbance and lower limb weakness, preventing him from walking unaided (MP4 21 MB)

Supplementary Material 3 (DOCX 25 KB)

## Data Availability

The data presented in this study can be obtained from the corresponding author upon reasonable request.
