# Supplementary material for: Multi-Omics Characterization of a KIF1C Structural Variant in a Patient with a Complex Movement Disorder Partially Responsive to Deep Brain Stimulation
Source: Cerebellum. 2026 Mar 24;25(2):41. doi: 10.1007/s12311-026-01963-x (PMC13013209; doi:10.1007/s12311-026-01963-x)
Supplement: Supplementary file 3 — Supplementary Material 3 (DOCX 25 KB) [file 12311_2026_1963_MOESM3_ESM.docx]

**Supplementary Table 1:** Genotype, demographics and phenotype of patients with pathogenic biallelic variants in the *KIF1C* gene reported in the literature

| **Reference** | **n** | **Family** | **Genotype** | | | **Demographics** | | | | | **Phenotype** | | | | |
| --- | --- | --- | --- | --- | --- | --- | --- | --- | --- | --- | --- | --- | --- | --- | --- |
|  |  |  | **Pathogenic**  **variants** | **Zygosity** | **Category** | **Descent** | **Sex** | **AAE**  **(y)** | **AAO**  **(y)** | **DD**  **(y)** | **Spasticity** | **Ataxia** | **Dystonia** | **Tremor** | **Additional symptoms** |
| Novarino et al.  Doi:10.1126/  science.1247363 | 3 | 1 | c.183G>A | Hom | Splice site variant | NA | NA | NA | NA | NA | Yes | NA | NA | NA | Yes (com-plicated HSP) |
|  |  | 2 | c.2191C>T,  p.Arg731* | Hom | Nonsense variant | NA | NA | NA | NA | NA | Yes | No | No | No | No (un-complicated  HSP |
|  |  | 3 | Deletion exon 14-18 | Hom | CNV | NA | NA | NA | NA | NA | Yes | NA | NA | NA | Yes (com-plicated HSP) |
| Dor et al.  Doi:10.1136/jmed genet-2013-102012 | 7 | 1 (V_1) | c.2191C>T, p.Arg731* | Hom | Nonsense variant | Palestinian | M | NA | 10 | NA | Yes (+) | Yes Dysarthria (++), Dysmetria (+++) | NA | Yes | Head titubation (++) |
|  |  | 1 (V_2) | c.2191C>T, p.Arg731* | Hom | Nonsense variant | Palestinian | M | NA | 13 | NA | Yes (++) | Yes Dysarthria (+++), Dysmetria (+++) | NA | Yes | Head titubation (++) |
|  |  | 1  (V_4) | c.2191C>T, p.Arg731* | Hom | Nonsense variant | Palestinian | F | NA | 10 | NA | Yes (+) | Yes Dysarthria (++), Dysemetria (+++) | NA | Yes | Head titubation (++) |
|  |  | 1  (V_6) | c.2191C>T, p.Arg731* | Hom | Nonsense variant | Palestinian | M | NA | 6 | NA | Yes (++) | Yes Dysarthria (+++), Dysmetria (+++) | NA | Yes | Head titubation (++) |
|  |  | 2 (II_1) | c.505C>T, p.Arg169Trp | Hom | Missense variant | Moroccan | M | NA | 7 | NA | No (-) | Yes  Ataxic gait (++) | Yes Cervical dystonia | Yes | Head titubation (+++) |
|  |  | 2 (II_5) | c.505C>T, p.Arg169Trp | Hom | Missense variant | Moroccan | F | NA | 16 | NA | No (-) | Yes Dysmetria (+), Ataxic gait (+) | NA | NA | Decreased visual acuity |
|  |  | 2 (II_7) | c.505C>T, p.Arg169Trp | Hom | Missense variant | Moroccan | M | NA | 1 | NA | No (-) | Yes Dysarthria (+), Dysmetria (+), Ataxic gait (+) | NA | NA | NA |
| Marchionni et al.  Doi:10.7916/tohm. v0.641 | 2 | 1 | c.1019+1dup | Hom | Splice site variant | Moroccan | M | 17 | ES | NA | Yes | Yes (SARA 12/40) | Yes Dystonic tremor | Yes | Asympto-matic demyeli-nating neuropathy |
|  |  | 1 | c.1019+1dup | Hom | Splice site variant | Moroccan | F | 17 | ES | NA | Yes | Yes | Yes | Yes | Asympto-matic demyeli-nating neuropathy |
| Yücel-Yılmaz et al.  Doi: 10.1016/j.braindev.  2018.02.013 | 3 | 1 (Family A, II:1) | c.463C> T, p.Arg155* | Hom | Nonsense variant | Turkish | M | 23 | 3 | 20 | Yes (+) | Yes  Truncal ataxia (+), Dysarthria (+), Dysmetria (+) | Yes | Yes | Titubation (+) |
|  |  | 1 (Family A, II:2) | c.463C> T, p.Arg155* | Hom | Nonsense variant | Turkish | M | 17 | 4 | 13 | Yes (+) | Yes  Truncal ataxia (+), Dysarthria (+), Dysmetria (+) | NA | Yes | Titubation (+) |
|  |  | 2 (Family B, II.1) | c.2478delA, p.Ala828Argfs*13 | Hom | Frame-shift variant | Turkish | F | 22 | 3 | 19 | No (-) | Yes  Truncal ataxia (+), Dysarthria (+), Dysmetria (+) | NA | Yes | Titubation (+) |
| Caballero Oteyza et al.  Doi:10.1212/WNL.0000000000000479 | 4 | 1 (THI26001-3) | c.305G>C, p.Gly102Ala  c.527C>T, p.Pro176Leu | Comp het | Missense variants | German | M | 48 | 18 | 30 | Yes Lower limb (+++), Upper limb (-) | Yes  Limb ataxia, intetion tremor, mild dysarthria | No (-) | Yes Intention tremor, irregular head tremor | NA |
|  |  | 1 (THI26001-4) | c.305G>C, p.Gly102Ala  c.527C>T, p.Pro176Leu | Comp het | Missense variants | German | M | 45 | 30 | 15 | Yes Lower limb (++), Upper limb (-) | Yes  Limb ataxia, intention tremor, mild dysarthria | No (-) | Yes Intention tremor) | Demyelin-ating sensor-motor neuropathy |
|  |  | 1 (THI26001-5) | c.305G>C, p.Gly102Ala  c.527C>T, p.Pro176Leu | Comp het | Missense variants | German | F | 42 | 18 | 24 | Yes Lower limb (+++), Upper limb (+) | Yes  Limb ataxia, intention tremor, moderate dysathria | Yes  Mild cervical dystonia | Yes Intention tremor | NA |
|  |  | 2 (IHG25125-5) | c.901A>G, p.Arg301Gly | Hom | Missense variant | Turkish | M | 48 | 10 | 38 | Yes Lower limb (+++), Upper limb (-) | NA  No limb ataxia, no intention tremor, but mild dysathria | No (-) | No (-) | NA |
| Laurie et al.  Doi: 10.1038/s41591-024-03420-w; Supplementary table 4 | 1 | 1 (FAM0003876) | c.1020-2A>G | Hom | Splice site variant | NA | F | NA | NA | NA | NA | Yes (Ataxia, Dysarthria) | NA | NA | NA |

n = Number of individuals; AAE – age at examination; y – years; AAO – age at onset; DD – disease duration; NA – not applicable; HSP – Hereditary Spastic Paraplegia; M – male; F – female; CNV – copy number variation; Hom – homozygous; Comp het – compound heterozygous.
